# A case of anesthesia mumps that required postoperative re-intubation

**DOI:** 10.1186/s40981-018-0159-0

**Published:** 2018-02-26

**Authors:** Takayuki Hamaguchi, Naho Suzuki, Ichiro Kondo

**Affiliations:** Department of Anesthesiology, Jikei University School of Medicine, Nishi-shinbashi 3-19-18, Minato-ku, Tokyo, Japan

**Keywords:** Anesthesia mumps, Salivary gland swelling, Parotid gland swelling, Upper airway obstruction

## Abstract

We encountered a 59-year-old man who first underwent left internal carotid endarterectomy for left internal carotid artery stenosis and then presented with postoperative swelling of the bilateral salivary glands. He then developed upper airway obstruction that required emergency tracheal intubation. The most likely cause was thought to be anesthesia mumps, which involves a complex interaction of multiple factors including pneumoparotitis, venous congestion, and excess saliva secretion. Many cases of salivary gland swelling recover after follow-up observation alone if there are no inflammatory findings; however, severe complications may sometimes occur. If upper airway obstruction develops as in the present case, then emergency airway management must also be considered and conscientious observation is necessary.

## Background

Anesthesia mumps consists of acute and transient salivary gland swelling caused by general anesthesia. It is an extremely rare postoperative complication and occurs following various surgical procedures. It is usually a self-limiting disease and requires only follow-up observation [1, 2], and in few case reports, evaluation with imaging studies have been performed.

We encountered a patient who required emergency tracheal intubation due to upper airway obstruction after anesthesia mumps following an internal carotid artery (ICA) endarterectomy (CEA). We include discussion on imaging evaluation—since preoperative and postoperative neck computed tomography (CT) images were available for this patient partly because neck surgery was performed—and a brief literature review.

## Case presentation

**Fig. 1:**
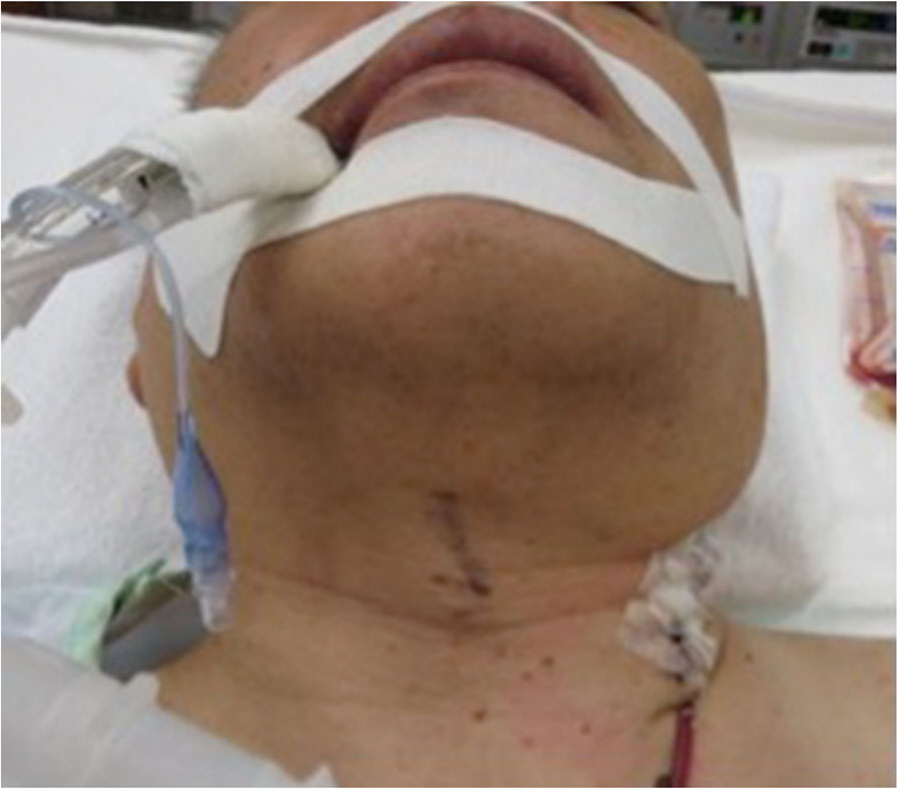
Photograph showing the swelling of his neck 7 h after the operation

**Fig. 2:**
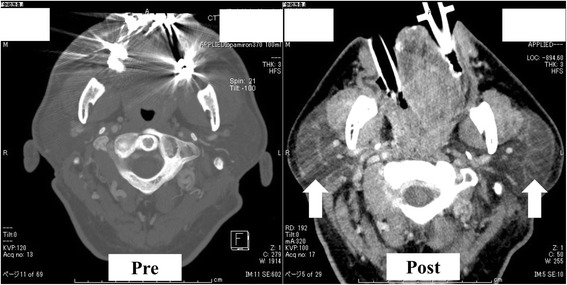
Post-operative image of computed tomography scans at the parotid glands level shows the bilateral parotid glands were swollen compared to the pre-operative state

**Fig. 3:**
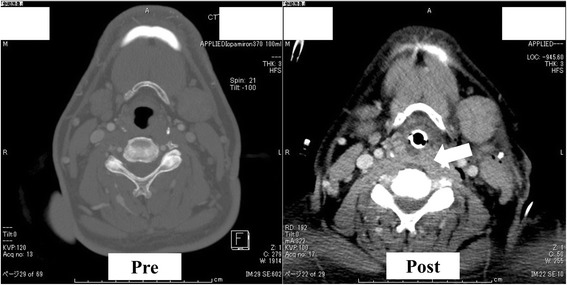
Post-operative image of computed tomography scans shows the edema of the posterior pharyngeal wall was developed compared to the pre-operative state

A 59-year-old man (170 cm, 78 kg) had cerebral infarction of the right precentral gyrus 2 years prior presentation and was diagnosed with bilateral ICA stenosis. He was treated conservatively (oral cilostazol and clopidogrel) and followed up; however, the stenosis of the left ICA progressed. Therefore, he was scheduled for CEA. There were no apparent complications related to his previous cerebral infarction. He was receiving an oral treatment for hypertension and dyslipidemia and had a long history of smoking (40 cigarettes per day for 40 years). There were no other notable findings in preoperative examinations. On the day of surgery, he was admitted to the operating room without premedication. Pre-oxygenation was performed using a mask without headband. Anesthesia was induced using 200 μg fentanyl and 5 mg midazolam, and 60 mg rocuronium was given to facilitate tracheal intubation. A Macintosh laryngoscope was used to expose the larynx. The view was classified as Cormack III. We attempted a tracheal intubation with a endotracheal tube (Shiley™ Endotracheal Tube with TaperGuard™ Cuff 7.5 mm), but the esophagus was inadvertently intubated, so it was removed. The second tracheal intubation by using a Macintosh laryngoscope was successful. Wheals appeared on the upper limbs, neck, and precordium without any vital sign changes after infusion of cefazolin sodium as a preoperative antibiotic, so administration was discontinued. The skin signs were believed to be an allergic reaction to cefazolin, which was changed to fosfomycin, and the patient also received an intravenous infusion containing 200 mg of hydrocortisone sodium phosphate. During surgery, he was placed in the supine position, but the neck was slightly rotated and lateroflexed to the right to secure the surgical field. Anesthesia was maintained with 1.4% sevoflurane and remifentanil at a dose of 0.2 μg/kg/min. Mild hypotension was observed intraoperatively. This was managed with fluid infusions and continuous administration of an appropriate dose of noradrenaline. Surgery was concluded without problems, and the preoperatively observed wheals resolved. Endotracheal aspiration was performed after waking up the patient, but it triggered a strong cough reflex. The patient was then extubated. The duration of surgery was 4 h and 57 min, the duration of anesthesia was 6 h and 29 min, the volume of blood loss was negligible, the in-out balance was + 3210 mL (crystalloid fluid 4175 mL, urine volume 965 mL), and mild swelling of the face and both upper limbs was noted. The patient was admitted to the intensive care unit (ICU) in a lucid state with a blood pressure of 120/70 mmHg, a heart rate of 110 beats/min, oxygen saturation of 100% on 3 L of nasal oxygen and respiratory rate of 12 breaths/min. His arterial blood gas analysis was normal, and mild hoarseness was noted without swelling of the neck or stridor. Six hours after admission to the ICU mild bilateral neck swelling appeared. The hoarseness rapidly worsened, and tachypnea and stridor appeared 1 h later without oxygen desaturation. Emergency intubation with a tube (Shiley™ Evac Endotracheal Tube with TaperGuard™ Cuff 7.5 mm) was immediately performed with mild sedation with propofol under spontaneous breathing on suspicion of upper airway obstruction. We used a video laryngoscope (HOYA Co. Ltd., airway scope) with a gum elastic bougie tube introducer. Laryngopharyngeal findings at this time included no epiglottic edema. However, the edema of the lateral and posterior pharyngeal walls was present, and it narrowed the oral and pharyngeal cavities. The neck swelling was pronounced, and the neck circumference was 63 cm (Fig. 1). A CT scan was performed to investigate the cause and to facilitate differential diagnosis of possible postoperative hemorrhage, but no bleeding was observed. The bilateral parotid and salivary glands were markedly swollen (Fig. 2), and the edema of the posterior pharyngeal wall was developed (Fig. 3) when compared to the preoperative state. Blood test results revealed a leukocyte count of 8000/μL (eosinophils 0%), amylase at 1790 U/L, mumps immunoglobulin G (IgG) at 16.6 (+), mumps IgM at 0.27 (−), and C-reactive protein at 0.18. Based on these findings, we concluded that the patient had previously suffered from a mumps infection, but had no active disease. Moreover, he only presented with the clinical features of sialadenitis. We commenced administration of steroids to reduce the edema. On postoperative day (POD) 2, the serum amylase decreased to 457 U/L, gradual improvement of the bilateral parotid gland swelling was observed, and the patient was extubated. The subsequent clinical course was favorable, and the neck swelling disappeared. The general condition, airways, and ability to swallow were all normal when the patient was discharged on POD 11.

## Conclusions

In this case, emergency intubation was performed because upper airway obstruction strongly suspected due to expiratory stridor and rapidly worsen dyspnea. His neck was bilaterally swelling and CT showed the bilateral parotid glands swelling and the edema of the posterior pharyngeal wall without hematoma around surgical site. Blood test indicated serum amylase was abnormally elevated. Thus, we diagnosed upper airway obstruction due to the anesthesia mumps. Furthermore, he developed an allergic reaction, which might have caused or intensified the edematous changes in the pharyngeal wall. This might be one of the etiologies that caused upper airway obstruction.

Anesthesia mumps is considered to be an extremely rare complication. According to reports in the literature, its occurrence is highly variable (0.2–17%) [3–5], and the exact incidence remains unknown. Although patients can develop postoperative salivary gland swelling, it is typically mild, with few subjective symptoms. Therefore, many cases resolve spontaneously without being noticed. Thus, we believe that the discrepancies in the incidence rate are largely due to variable amount of attention that is paid to this condition.

The cause and detailed pathophysiology of anesthesia mumps remains unknown, but salivary duct occlusion, sialorrhea, venous congestion and venostasis, involvement of the autonomic nerves, and side effects of medications have been suggested [5, 6].

Causes of salivary gland obstruction include (1) physical compression by lateral position, positions that rotate and flex the neck, compression by endotracheal tubes, mucosal lesions and edema; (2) pneumoparotitis induced by the penetration of air as a result of retrograde flow into the salivary duct due to positive pressure in the oral cavity by mask ventilation, pharyngeal reflex, and cough reflex; and (3) dehydration, administration of atropine, and sympathetic nervous system activation due to invasiveness of surgery causing increased salivary viscosity, which may in turn itself cause an occlusion.

Salivation is mediated by the autonomic nerves, and the main secretory innervation is parasympathetic. Intratracheal manipulation stimulates parasympathetic nerves that mediate the pharyngeal reflex, which promotes salivation and leads to vasodilation and hyperemia in the salivary gland [2]. Stimulation of the sympathetic nerves also evokes salivation, but it is relatively short-lasting, the saliva is usually thick and mucinous, and vasoconstriction occurs [7]. Actually, noradrenaline infusion increases salivary alpha-amylase, a digestive enzyme secreted from the salivary glands that has been proposed as a sensitive surrogate maker for activity stress [8].

In the present case, we observed bilateral swelling of the parotid and submandibular glands. Therefore, we considered it unlikely that there was only a mechanical regional occlusion related to patient positioning and pneumoparotitis. We believe that variety of causes led to development of anesthesia mumps. One of them is a venous congestion due to surgical procedure or stimuli of CEA, and the others are excess secretion of saliva due to reflex salivation, the pharyngeal reflex or continuous administration of noradrenaline. However, the exact causes remain unclear.

Several reports state that most cases of anesthesia mumps resolve spontaneously with follow-up observation alone, it has been considered that rehydration therapy and anti-inflammatory drugs if needed are sufficient to treat it [1, 2]. However, there may be cases, such as the present case, in which patients suffer from severe complications, including upper airway obstruction [9, 10]. In our patient, the combined approach of a video laryngoscope and a gum elastic bougie tube introducer resulted in a successful re-intubation without the need for tracheostomy. Emergency tracheostomy might be often chosen because of difficult mask ventilation and the possibility of intubation difficulty for postoperative dyspnea and acute airway obstruction [10]. However, in cases involving massive edema and swelling of the neck, it is difficult to identify the location of the tracheostomy. Video laryngoscopes could well become the standard procedure for these types of patients who need emergency tracheal intubation. Hence, additional measures to treat these conditions are required, and conscientious follow-up observation is necessary.

## Abbreviations

CEA: Carotid endarterectomy
CT: Computed tomography
ICA: Internal carotid artery
ICU: Intensive care unit
POD: Postoperative day
